# Sequence analysis of *Plasmodium falciparum *cytochrome b in multiple geographic sites

**DOI:** 10.1186/1475-2875-6-164

**Published:** 2007-12-17

**Authors:** Marie-Thérèse Ekala, Nimol Khim, Eric Legrand, Milijaona Randrianarivelojosia, Ronan Jambou, Thierry Fandeur, Didier Menard, Serge-Brice Assi, Marie-Claire Henry, Christophe Rogier, Christiane Bouchier, Odile Mercereau-Puijalon

**Affiliations:** 1 Immunologie Moléculaire des Parasites, CNRS URA 2581, Institut Pasteur, 25 rue du Dr ROUX, 75724 Paris Cedex 15, Paris, France; 2 Unité d'Epidémiologie Moléculaire, Institut Pasteur du Cambodge, Phnom Penh, Cambodia; 3 Laboratoire CNRCP Cayenne, Institut Pasteur de la Guyane, French Guiana; 4 Unité de Recherche sur le Paludisme, Institut Pasteur de Madagascar, Antananarivo, Madagascar; 5 Laboratoire d'Immunologie Parasitaire, Institut Pasteur de Dakar, Dakar, Senegal; 6 Institut Pierre Richet, Bouaké, Ivory Coast; 7 Institut de Médecine Tropicale du Service de Santé des Armées, Marseille, France; 8 Plate-forme Génomique – Pasteur Génopole Ile de France, Institut Pasteur, Paris, France

## Abstract

**Background:**

The antimalarial drug atovaquone specifically targets *Plasmodium falciparum *cytochrome b (*Pfcytb*), a mitochondrial gene with uniparental inheritance. Cases of resistance to atovaquone associated with mutant *Pfcytb *have been reported, justifying efforts to better document the natural polymorphism of this gene. To this end, a large molecular survey was conducted in several malaria endemic areas where atovaquone was not yet in regular use.

**Methods:**

The polymorphism of the *Pfcytb *was analysed by direct sequencing of PCR products corresponding to the full length coding region. Sequence was generated for 671 isolates originating from three continents: Africa (Senegal, Ivory Coast, Central African Republic and Madagascar), Asia (Cambodia) and South America (French Guiana).

**Results:**

Overall, 11 polymorphic sites were observed, of which eight were novel mutations. There was a large disparity in the geographic distribution of the mutants. All isolates from Senegal, Central African Republic and Madagascar displayed a Camp/3D7 wild type *Pfcytb *sequence, as did most samples originating from Cambodia and Ivory Coast. One synonymous (t759a at codon V253V) and two non-synonymous (t553g and a581g at codons F185V and H194R, respectively) singletons were detected in Ivory Coast. Likewise, two synonymous (a126t and c793t at codons -T42T and L265L, respectively) singletons were observed in Cambodia. In contrast, seven mutated sites, affecting seven codons and defining four mutant haplotypes were observed in French Guiana. The wild type allele was observed in only 14% of the French Guiana isolates. The synonymous c688t mutation at position L230L was highly prevalent; the most frequent allele was the c688t single mutant, observed in 84% of the isolates. The other alleles were singletons (a126t/a165c, a4g/a20t/a1024c and a20t/t341c/c688t corresponding to T42T/S55S, N2D/N71I/I342L, N71I/L114S/L230L, respectively" please replace with ' corresponding to T42T/S55S, N2D/N71I/I342L and N71I/L114S/L230L, respectively). The codon 268 polymorphisms associated with atovaquone resistance were not observed in the panel the isolates studied. Overall, the wild type PfCYTb protein isoform was highly predominant in all study areas, including French Guiana, suggesting stringent functional constraints.

**Conclusion:**

These data along with previously identified *Pfcytb *field polymorphisms indicate a clustering of molecular signatures, suggesting different ancestral types in South America and other continents. The absence of mutations associated with most atovaquone-proguanil clinical failures indicates that the atovaquone-proguanil association is an interesting treatment option in the study areas.

## Background

A high rate of treatment failure for commonly anti-malarial drugs used in *Plasmodium falciparum *infections has been reported in numerous endemic areas. The recommended treatment policy is now to use drug combinations [1]. The atovaquone-proguanil (AP) drug combination, distributed under the trade name of Malarone^®^, is one of the treatment and prophylaxis options. AP has a high cure rate, limited mild side effects [2] and proved efficacious against multi-drug resistant *P. falciparum *malaria [3,4]. Atovaquone (a hydroxy-naphthoquinone) is a potent inhibitor of the cytochrome bc1 (cytbc1) complex [5-8], a key respiratory enzyme from the mitochondrial membrane, while proguanil (an isopropylbiguanide) inhibits the plasmodial dihydrofolate reductase [4,9,10]. The association synergizes to collapse the mitochondrial membrane [8,11].

*Plasmodium falciparum in vitro *resistance to atovaquone has been associated with specific point mutations in the *cytochrome b *gene (*Pfcytb*) in the region spanning codons 271–284 [7,12]. A high frequency of recrudescence was observed in patients receiving atovaquone as a single drug therapy against *P. falciparum *[13,14]. A Y268S point mutation in the *Pfcytb *gene, distinct from the mutations observed in the lines selected *in vitro *for atovaquone resistance, was detected in the recrudescing parasites [7]. Codon 268 polymorphism was used as marker for a molecular surveillance of atovaquone-proguanil resistance [15-19]. AP treatment failures were increasingly reported a few years after its introduction, with recrudescing parasites presenting a markedly increased IC_50 _for atovaquone [15,20,21]. In most cases, recrudescence was associated with a mutant 268 codon, either a Y268S [3,12,20,22-26], a Y268N [15] or a Y268C mutation [21]. However, the presence of a mutant 268 codon was not observed in all cases of AP failure [21,27].

Along with the key issue of emergence and spreading of polymorphisms conferring atovaquone resistance, analysis of *Pfcytb *field diversity presents an interest in population genetics [28-31]. Indeed, the *cytb *gene is encoded by the mitochondrial DNA and as a consequence, is of uniparental inheritance and under quite different evolution constraints compared to nuclear genes [32,33]. In particular, interallelic recombination is not possible and polymorphisms such as base substitutions or insertions may accumulate over time. In mammals, the *cytb *locus displays an approximately 10-fold higher mutation rate than nuclear genes [34]. A rapid mutation rate (one mutation in 10^5 ^parasites) was reported for *Pfcytb *[35], but 100–1,000 lower rates were described subsequently [7].

Sequence polymorphism of the near to full gene sequence has been explored in laboratory [7,36] and *in vitro *resistant isolates [7,12]. It has also been looked for in cases of treatment failures from several countries, mostly African countries [7,12,15,21,22,24-26]. Systematic analysis of field polymorphism in African settings [37,38] and in isolates from the Thai Myanmar border [39] essentially focused on the region coding for the atovaquone-binding site. Full gene sequence analysis of field samples has been restricted to isolates from few patients in India [36] and from patients returning to France from West Africa, Central Africa or the Indian Ocean [21,25,40]. This provides an interesting picture of the overall polymorphism of the gene, but little clues on possible population signatures related to this gene. Sequence analysis of mitochondrial DNA from 100 independent isolates   collected worlwide provided evidence for geographical clustering [29,30].

To further document *Pfcytb *population polymorphism, 671 isolates from six different areas: Africa (Senegal, Ivory Coast, Central African Republic, and Madagascar), Asia (Cambodia) and South America (French Guiana) were AP had not been in regular use were analysed. This identified numerous novel, low frequency polymorphisms in Africa and Cambodia, most of which were country-specific, together with a high frequency signature that was specific for the South American isolates. None of the polymorphisms previously associated with atovaquone resistance *in vitro *or AP treatment failure was observed in this panel of isolates.

## Methods

### Study-sites and sample collection

Isolates were collected during the drug susceptibility surveillance programme conducted by the reference laboratory based in French Guiana, as such they were exempt from consent. Additional isolates originating from Senegal [41], Madagascar, Cambodia [42], Ivory Coast [43] and Central African Republic [44] were collected from patients recruited at home or at health centres during regular control surveys. Informed consent was obtained for these studies.

Blood samples were collected in each country from patients with mild malaria in the years 2000–2003, except in Central African Republic where blood was collected in the year 2004. After blood smear analysis, patients with mixed species infections were excluded. Only patients with positive slides for *P. falciparum *were included (Senegal : Sn, N = 45 ; Madagascar : Mg, N = 192 ; Ivory Coast : IC, N = 44 ; Cambodia : Kh, N = 179 ; French Guiana : FG, N = 160 ; Central African Republic : RCA, N = 51). Blood was stored frozen before being processed for genomic DNA isolation and amplification.

### DNA extraction and Polymerase Chain Reaction (PCR) amplification

Parasite DNA was extracted from frozen blood aliquots using the phenol/chloroform method [45]. Amplifications were performed in 50 μL final reaction volume containing DNA template, 1 μM each primer, 200 μM each dNTP, 1.75 mM MgCl2 and 2.5 U Taq polymerase (Promega) using a Mastercycler Gradient 5331, Eppendorf. The primers were designed to amplify the full length *Pfcytb *gene, using as reference sequence the atovaquone-sensitive *P. falciparum *3D7 clone (accession No AY282930), and carried an extension sequence (bold) that was used as sequencing primer as well: *cytb1*-sense (**ctcgaggaattcggatcc**tatgaacttttactctattaatt) and *cytb2*-antisense (**tctagaaagcttggatcc**tatatgtttgcttgggagct). The PCR amplification conditions were: 1 cycle denaturation at 94°C for 3 min, followed by 5 cycles [94°C for 30 sec, 56°C for 90 sec, 65°C for 150 sec] and 35 cycles [94°C for 10 sec, 53°C for 90 sec, 65°C for 150 sec]. A final extension was done at 65°C during 15 min. The isolates from Ivory Coast were amplified using the primers CYTb1 and CYTb2 [12].

### Direct sequencing of PCR products

The PCR products were purified using a P-100 Gel Fine solution (Bio-Rad) and Multiscreen MAVN45 kit system (Millipore). The amount of PCR product (1131 bp) was estimated on a 1.2% agarose gel. Sequencing reactions were performed on both strands using the extension primers (sequence in bold indicated above) and internal primers using ABI Prism *BigDye Terminator *chemistry. Sequencing reactions conditions were as follows: 1 cycle at 96°C for 60 sec, followed by 25 cycles [96°C for 10 sec, 50°C for 5 sec, 60°C for 4 min]. The product was ethanol precipitated and washed with 70% Ethanol. The pellets were resuspended in 10 μL 0.3 mM EDTA and sequenced using an ABI PRISM 3100 Genetic analyzer (Applied Biosystems).

The isolates from Ivory Coast were sequenced on both strands with the internal primers PfCYTB33 (5'atttatgatatttattgtaactgc) and PfCYTB4R (5'agttggttaaacttctttgttctgc), covering codons 122 to 294 (i.e. encompassing the binding site of atovaquone).

### Data analysis

Sequence analysis was done using Phred Phrap consed package [46]. Sequences with segments ≥ 1000 bp called with a quality over 20 per base were retained. Only unambiguous single nucleotide polymorphisms (SNPs) were considered. Sequences of insufficient quality were either resequenced or rejected. The sequence assembly was done with the Seqscape software v.2.0. (Applied Biosystems).

## Results

**Figure 1:**
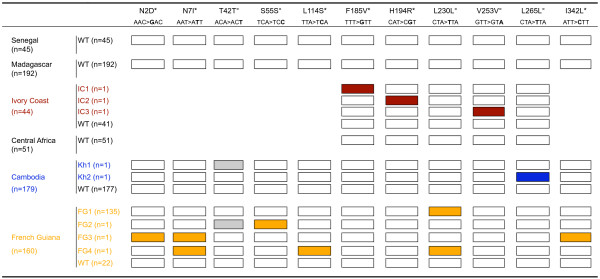
**Individual *Pfcytb *polymorphisms and allelic types observed in the study areas**. WT : wild type ; * non synonymous mutation; ° synonymous mutation. The polymorphic sites observed in this study are shown by codon (numbers refer to positions in the protein sequence). Amino acids are indicated with single letter code and in capital, the nucleotide changes are indicated below (bold). Area-specific mutations are coloured in brown, blue and orange for Ivory Coast, Cambodia and French Guiana, respectively. In grey, T42T, the only mutation found common between two settings.

**Figure 2:**
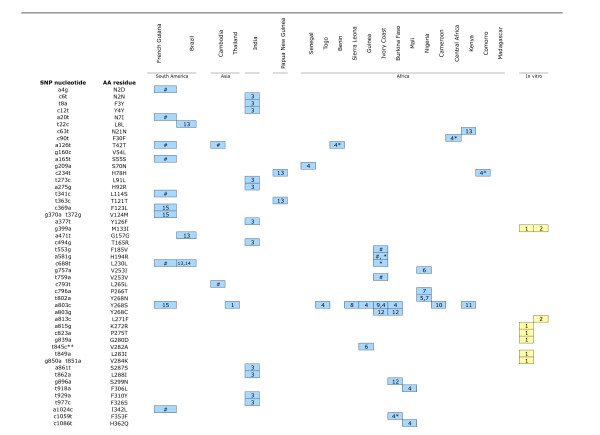
**Compilation of the polymorphic sites of the *Pfcytb *gene in field isolates (in blue) and in lines selected for atovaquone-resistance in vitro (in yellow)**. The figure does not include silent mutations, which were not described [36] or whose nucleotide numbering does not match with the reported reference sequences [37, 40]. Numbering indicated refers to data reported from 1-Korzinczky et al [7]; 2-Schwöbel et al. [12]; 3-Sharma et al. [36]; 4-Musset et al. [25]; 5-Fivelman et al. [15]; 6-Berry et al. [40]; 7-Happi et al. [38]; 8-Kuhn et al. [24]; 9-Färnert et al. [23]; 10-David et al. [22]; 11-Schwartz et al. [26]; 12-Musset et al. [21]; 13-Joy et al. [30]; 14-Conway et al. [29]; 15-Legrand et al. [20]. # Single Nucleotide Polymorphism found in this study; * Musset personal communication; ** codon 282 is GTA : nucleotide 846 is an A and not a C as quoted by Berry et al [40], we assume that the T to C mutation affects nucleotide 845

Among the 671 isolates, a full length *Pfcytb *sequence was successfully established for 576 isolates and partial sequence was obtained for 95 isolates (Figure 1). Overall, 11 polymorphic sites were observed (Figure 1), of which eight were novel mutations compared with published data (Figure 2).

All isolates from Senegal, Central African Republic and Madagascar had the same sequence, which was identical to the Camp/3D7 reference sequence. One synonymous point mutation and two coding mutations were observed in the set of isolates from Ivory Coast (Figure 1). None of these had been published previously and each was observed in a single isolate.

In Cambodia, two synonymous mutations (T42T and L265L) were observed (Figure 1). Each was detected in one isolate, the remaining 177 isolates harboured the Camp/3D7 reference *Pfcytb *gene sequence.

**Figure 3:**
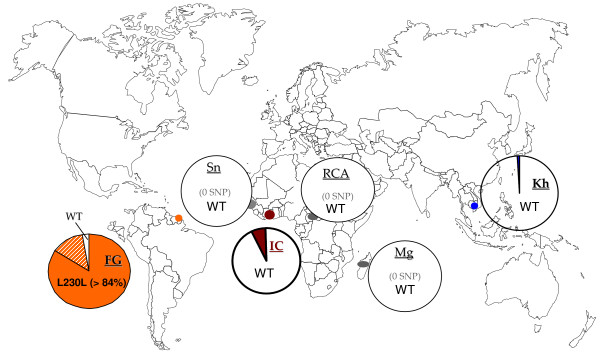
**Geographic distribution of the *Pfcytb *gene polymorphism studied here**. Country's code: FG: French Guiana, Sn: Senegal, IC: Ivory Coast, RCA: Central Africa, Mg: Madagascar, Kh: Cambodia. Allelic frequency in each site is depicted in colour coded pie charts: brown, blue and orange referring to Ivory Coast, Cambodia and French Guiana, respectively. The wild type allele (WT) is indicated in white.

The French Guiana samples presented the largest polymorphism, with seven mutated sites affecting seven codons. Five alleles were observed (Figure 1). Unlike the other settings where alleles had a single mutated position, three out of the five alleles from French Guiana were multiple mutants with two (allele FG2) or three (alleles FG3 and FG4) mutated codons. The Camp/3D7 reference allele was present in 22 out of 160 isolates (14%). Allele FG1, which carried a silent L230L mutation, was highly predominant, accounting for 135 of 160 (84%) isolates (Figures 1 and 3). The same mutation was associated with additional SNPs in allele FG4, which therefore probably derive from FG1. Allele FG4 which carried additional mutations N7I and L114S, was observed only once (frequency 0.6%). The N7I polymorphism was detected in allele FG3 as well, but in this case it was associated with N2D and I342L (Figure 1).

## Discussion

In the panel of isolates studied here, 11 polymorphic sites were observed, resulting in 11 codons displaying a single point mutation. Ten distinct alleles could be identified, as shown in Figure 1. Apart from the a126t and c688t silent mutations at codon T42T and L230L, respectively, and a581g non synonymous mutation at codon H194R, all mutations observed here were novel (Figures 1 and 2). Five synonymous and six non-synonymous mutations were detected. The observed ratio of synonymous to non synonymous mutations is in line with four synonymous and five non synonymous mutations detected in 270 full gene sequences of isolates from West Africa and the Indian Ocean [25] and four synonymous and six non synonymous mutations in 14 isolates from India [36], but lower than the three to one ratio observed in Gabon [37] and the nine to three ratio reported for 135 isolates from West and Central Africa [40]. The compilation of all *Pfcytb *field polymorphisms identified so far (Figure 2) highlights 50 polymorphic sites, affecting 45 codons, with 30 mutant amino acid residues.

There was a clear geographical heterogeneity in the level and type of polymorphism (Figure 3). The Camp/3D7 type was the single allelic form detected in three African settings studied here (Madagascar, Senegal and Central African Republic). Similar observations were made in Ethiopia [37]. In Ivory Coast, the same allele was observed in 93% of the patients, a frequency similar to the 90% prevalence observed in Gabon [37], or in travellers returning to France from West and Central Africa [40]. As observed in other African endemic settings [37], the mutant alleles were single mutants and each mutant had a low frequency, being observed in one or two isolates. A similar low frequency of single mutant alleles has been observed in the panel of isolates from patients returning from travel to West and Central Africa and the Indian Ocean [25,40].

In Cambodia, the Camp/3D7 wild type allele was also highly predominant, consistent with recent sequence data of the atovaquone binding site in patients from the Thai/Myanmar border [39]. The two mutations observed were both synonymous and detected at low frequency.

French Guiana showed a quite different profile, and was the most polymorphic of the six countries explored. Seven of 11 mutant sites were observed in the set of isolates from French Guiana. Furthermore, this area was the only one where multiple mutant alleles were detected. Importantly, the Camp/3D7 wild type allele, which was dominant in the other settings was observed with a 14% frequency only, while the dominant allele (84% frequency) was a single, silent L230L mutant. This was in accordance with data from other localities from South America [29,30]. Triple mutants, most probably originating from the L230L parent for FG4 isolate, were observed along with a double mutant possibly derived from the Camp/3D7 type. Thus, the French Guiana parasite population had a *Pfcytb *pattern dissimilar from the African and Cambodian settings. This was consistent with data from others [29,30]. A specific, high frequency geographical signature seems to prevail in India as well, where 13 or 14 isolates carried a N2N F3Y Y4Y haplotype [36]. However, polymorphism in French Guiana was larger than in India, where three allelic forms have been reported. Thus, of all geographical areas studied so far, French Guiana presents the largest *Pfcytb *gene polymorphism. This is interpreted as a consequence of its population structure, with hypoendemic, isolated foci that are propitious to genetic drift [47].

**Figure 4:**
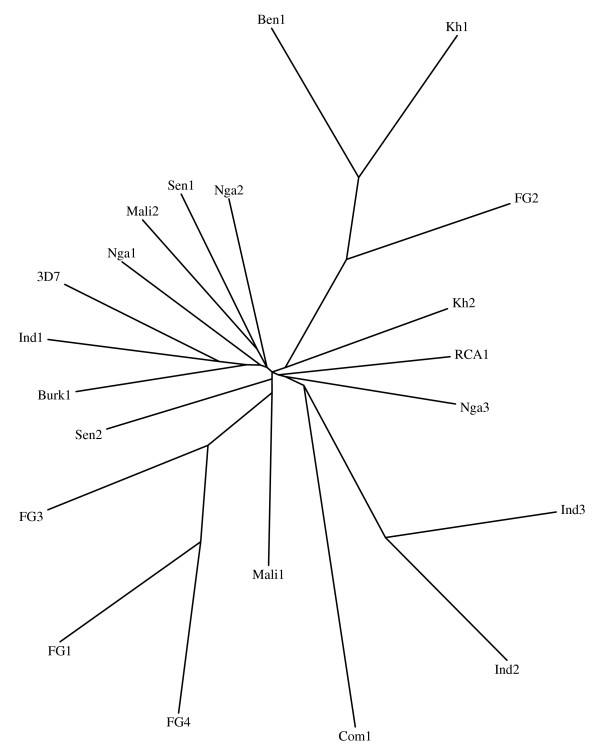
**Phylogenetic relationship among 20 *Pfcytb *alleles from various geographical origin**. The tree was estimated using the Phylip DNAdist program and the neighbour-joining method (100 replications), and drawn using Drawtree software. Among the various *Pfcytb *haplotypes previously published, only those corresponding to a natural polymorphism of the gene (i.e. not selected under atovaquone pressure) were considered for the phylogenetic analysis. The 3D7 sequence >gi|31789515|gb|AY282930 was used as reference. The haplotypes were as follows: Nga1: G757A [40]; Nga2: T802A, Nga3: C796A [38]; Sen1: G160C, Sen2: G209A, Mali1: T918A, Mali2: T1086A, Ben1: A126T, Com: C234T, Burk1: C1059T, RCA1: C90T [21]; Kh1: A126T, Kh2: C793T, FG1: C688T, FG2:A126T/A165C, FG3:A4G/A20T/A1024C; FG4:A20T/T341C/C688T (this paper); Ind1: T273C/A377T/C494G/A861T/T862A/T929A, Ind2: C6T/T8A/C12T/A275G, Ind3: C6T/T8A/C12T/A275G/T977C [36]. Codes used for countries: Senegal: Sen, Comoro islands: Com, India: Ind, Central Africa: RCA, Mali, Nigeria: Nga, French Guiana: FG, Benin: Ben, Burkina Faso: Burk, Kh: Cambodia.

*Pfcytb *nucleotide polymorphism was larger than the deduced protein sequence. All three alleles from Cambodia coded for the same, wild type protein sequence. In French Guiana the wild type deduced protein sequence accounted for 158 of 160 alleles. Thus in all geographic regions, the wild type protein sequence was the highly dominant if not the only predicted isoform. Altogether, these data point to an elevated mutation rate of the locus, with albeit a large dominance of the wild type protein sequence, probably indicating its optimal fitness. At the nucleotide level, interesting geographic clustering of molecular signatures were observed (Figure 4), suggesting different ancestral types in French Guiana (and in South America) as well as in India [36]. Specificities of the parasite population in India is also suggested by polymorphism of the *Pfcrt *locus [48]. The data reported here further support the evidence that the present parasite population from South America differs from the parasites from Africa, regarding surface antigens [49], loci such as *Pfcg2 *[31], *Pfcrt *[50-52], *Pfdhfr *and *Pfdhps *[51] and numerous genome-wide scattered SNPs [53]. The large *Pfcytb *polymorphism together with the observation that the *var *gene repertoire in Brazilian isolates is small and highly redundant unlike in Africa and Southeast Asia [54], raises the question of the structuring of the South American *P. falciparum *population.

None of the field polymorphism observed here or in other settings concerned the residues that are mutated in lines selected *in vitro *for atovaquone resistance [5,7,12]. Furthermore, no mutant Y268S, Y268C and Y268N *Pfcytb*, selected under atovaquone or AP pressure [3,7,12,15,21,22,24-26] was detected in any of the settings explored here. The 268N mutant, which has been observed with a 4.5% frequency in Nigeria in the absence of AP pressure [38] was not detected. This mutation has been reported so far essentially in Nigeria [15,38] and, as discussed by Happi *et al *[38] may have arisen under pressure by related drugs. The Y268S polymorphism was not detected in the isolates from French Guiana studied here, which were collected before implementation of AP as prophylaxis and as second line treatment in 2002. It was however observed one year after implementation in a second line AP-treatment failure [20], further substantiating the interpretation of its selection during AP treatment.

## Conclusion

Overall, the available data indicate an elevated mutation frequency of the *Pfcytb *locus, with multiple polymorphic sites, and in some cases more than one polymorphism per site, consistent with the elevated mutation frequency of mitochondrial genes [34,35]. The geographical clustering observed here adds further support to the evidence that the parasite population from South America differs from the parasites from Africa. Importantly, the absence of a mutated codon 268 in all settings investigated here indicates that AP remains an interesting treatment option for these areas. In view of the high mutation rate and of the rapid selection of mutants under AP pressure, careful surveillance of emerging *Pfcytb *mutants and resistance to atovaquone used as prophylaxis in travellers or as treatment is warranted.

## Authors' contributions

MTE did the sequencing of isolates except for Ivory Coast, was responsible for data collection, entry in the database and drafted the manuscript.

RJ (Senegal), MR (Madagascar), EL (French Guiana), DM (Republic Central Africa), SBA, MCH, CR (Ivory Coast) were responsible for sample collection and coordination of laboratory in the field.

NK, EL, MR, RJ, DM and CR  performed all PCR   amplifications and actively participated in sample collection.

TF established suitable protocols for *Pfcytb *amplification, conducted the phyologenetic study, participated in drafting the manuscript and was responsible for coordination of laboratory work in Cambodia.

CB was responsible for the sequencing procedure.

OMP conceived the study, helped for sequence analysis, drafted and revised the manuscript.

All authors read and gave the final approval of the version to be published.
